# Imaging and Risk Stratification in Pulmonary Arterial Hypertension: Time to Include Right Ventricular Assessment

**DOI:** 10.3389/fcvm.2022.797561

**Published:** 2022-03-25

**Authors:** Faisal Alandejani, Abdul Hameed, Euan Tubman, Samer Alabed, Yousef Shahin, Robert A. Lewis, Krit Dwivedi, Aqeeb Mahmood, Jennifer Middleton, Lisa Watson, Dheyaa Alkhanfar, Christopher S. Johns, Smitha Rajaram, Pankaj Garg, Robin Condliffe, Charlie A. Elliot, A. A. Roger Thompson, Alexander M. K. Rothman, Athanasios Charalampopoulos, Allan Lawrie, Jim M. Wild, Andrew J. Swift, David G. Kiely

**Affiliations:** ^1^Department of Infection, Immunity and Cardiovascular Disease, University of Sheffield, Sheffield, United Kingdom; ^2^Sheffield Pulmonary Vascular Disease Unit, Royal Hallamshire Hospital, Sheffield Teaching Hospitals NHS Foundation Trust, Sheffield, United Kingdom; ^3^Institute for in silico Medicine (INSIGNEO), University of Sheffield, Sheffield, United Kingdom; ^4^Norwich Medical School, University of East Anglia, Norwich, United Kingdom

**Keywords:** pulmonary hypertension, pulmonary arterial hypertension (PAH), right atrial area, right ventricular (RV), risk stratification, cardiac magnetic resonance imaging (cMRI)

## Abstract

**Background:**

Current European Society of Cardiology and European Respiratory Society guidelines recommend regular risk stratification with an aim of treating patients with pulmonary arterial hypertension (PAH) to improve or maintain low-risk status (<5% 1-year mortality).

**Methods:**

Consecutive patients with PAH who underwent cardiac magnetic resonance imaging (cMRI) were identified from the Assessing the Spectrum of Pulmonary hypertension Identified at a Referral centre (ASPIRE) registry. Kaplan–Meier survival curves, locally weighted scatterplot smoothing regression and multi-variable logistic regression analysis were performed.

**Results:**

In 311 consecutive, treatment-naïve patients with PAH undergoing cMRI including 121 undergoing follow-up cMRI, measures of right ventricular (RV) function including right ventricular ejection fraction (RVEF) and RV end systolic volume and right atrial (RA) area had prognostic value. However, only RV metrics were able to identify a low-risk status. Age (*p* < 0.01) and RVEF (*p* < 0.01) but not RA area were independent predictors of 1-year mortality.

**Conclusion:**

This study highlights the need for guidelines to include measures of RV function rather than RA area alone to aid the risk stratification of patients with PAH.

## Introduction

Pulmonary arterial hypertension (PAH) is a progressive life shortening condition. The 2015 European Society of Cardiology and European Respiratory Society (ESC/ERS) guidelines stratified patients with PAH into three groups; low-risk (<5%), intermediate-risk (5-10%), and high-risk (>10%) of 1-year mortality (1). Variables used in risk assessment include symptoms, exercise capacity, haemodynamics and imaging metrics. In PAH, failure of the right ventricle to cope with increased afterload results in haemodynamic changes with rises in right ventricular (RV) end diastolic pressure and right atrial (RA) pressure. Current guidelines include RA area but not measures of RV function to aid risk stratification. An extensive body of literature has shown that cardiac magnetic resonance imaging (cMRI) derived RV measurements are prognostic, predict clinical worsening (2–4), are sensitive to treatment effect (5) and have utility in risk stratification in PAH (6), however, there are limited data on RA area measurements (7, 8). To our knowledge no study has assessed the accuracy of RA area thresholds to assess risk of 1-year mortality. This study compares RV and RA area measurements and their utility to risk stratify patients with PAH, using the reference standard for assessment of cardiac structure and function, by comparing published thresholds for RV metrics (6) with current ESC/ERS thresholds for RA area (1).

## Methods

### Patients

Consecutive patients with PAH (idiopathic PAH, heritable PAH, and PAH in association with connective tissue disease, congenital heart disease, portal hypertension, human immunodeficiency virus infection, and drugs and toxins) who underwent cMRI between January 2008 and March 2017 were identified from the ASPIRE (Assessing the Spectrum of Pulmonary hypertension Identified at a Referral centre) registry, after a standardised systematic assessment (9). Patients were required to have mean pulmonary artery pressure ≥25 mmHg and pulmonary arterial wedge pressure ≤15 mmHg at right heart catheter. Incident treatment naïve patients who underwent a cMRI at baseline who had a further cMRI after a minimum of 3 months and before 31 March 2017 were included in the follow-up cohort. Ethical approval for this single centre study was obtained (ASPIRE, c06/Q2308/8).

### Cardiac Magnetic Resonance Imaging Acquisition and Image Analysis

Cardiac magnetic resonance imaging studies were completed using an 8-channel cardiac coil on a whole-body scanner at 1.5T GE HDx (GE Healthcare, Milwaukee, WI, United States), as previously described (6). Image analysis was performed on MASS software (MASS, research version 2020; Leiden University Medical Center, Leiden, Netherlands) with the observer blinded to the patient’s clinical information, cardiac catheter parameters and outcome data. RV endocardial and epicardial surfaces were identified using artificial intelligence software with manual correction as required, from the stack of short-axis cine images to obtain RV volumetric and functional measurements. RV trabeculations were included in the blood pool. RA endocardial surfaces were manually traced to measure maximum RA area at RV end systole. The RA appendage was manually excluded during contouring.

### Statistical Analysis

Data for continuous variables are displayed as mean ± standard deviation. Survival data were censored on 30 September 2020. Kaplan–Meier survival curves were evaluated using the log rank (Mantel–Cox) test. Locally weighted scatterplot smoothing (LOESS) regression analysis was performed to understand relationships between variables. Binary logistic regression was used to assess for predictors of 1-year mortality. A *p*-value < 0.05 was considered statistically significant.

## Results

### Patients

A total of 311 consecutive treatment-naïve patients were identified and 121 patients underwent follow-up cMRI at a mean interval of 1.9 ± 1.8 years. The mean age was 56.7 ± 16.1 years, and 72% of patients were female. The majority (82%) had idiopathic/heritable PAH (33%), PAH in association with connective tissue disease (49%) including 117 patients with systemic sclerosis (38%). Data for demographics, haemodynamics and cMRI metrics at baseline are shown in Table 1. The mean interval between the date of diagnosis and baseline cMRI (*n* = 311) was 1.2 ± 6.5 months and between the date of diagnosis and follow-up cMRI (*n* = 121) was 1.8 ± 1.7 years.

**TABLE 1:** Baseline demographics, haemodynamics, and cardiac MRI metrics.

	Baseline (*n* = 311)	IPAH/HPAH (*n* = 101)	PAH-CTD (*n* = 154)	PAH-CHD (*n* = 24)	PoPH (*n* = 22)	Other PAH (*n* = 10)
**Demographics**						
Age, year	56.7 ± 16.1	50.9 ± 17.6	63.2 ± 13.0	48.0 ± 18.1	53.5 ± 11.0	43.3 ± 6.1
Sex, F/M (F %)	223/88 (72)	70/31 (69)	119/35 (77)	20/4 (83)	12/10 (55)	2/8 (20)
WHO FC I, *n* (%)	1 (1)	0 (0)	0 (0)	1 (4)	0 (0)	0 (0)
WHO FC II, *n* (%)	14 (4)	5 (5)	4 (3)	2 (8)	2 (9)	1 (10)
WHO FC III, *n* (%)	261 (84)	76 (75)	137 (89)	20 (87)	19 (86)	9 (90)
WHO FC IV, *n* (%)	34 (11)	20 (20)	13 (8)	0 (0)	1 (5)	0 (0)
**PAH subtype, *n* (%)**						
IPAH/HPAH	101 (33)					
PAH-CTD	154 (49)					
PAH-CHD	24 (8)					
PoPH	22 (7)					
Other	10 (3)					
**Haemodynamics**						
mRAP, mm Hg	10 ± 6	12 ± 6	9 ± 6	11 ± 5	11 ± 7	15 ± 8
mPAP, mm Hg	48 ± 14	57 ± 12	41 ± 12	55 ± 8	46 ± 8	52 ± 6
PAWP, mm Hg	10 ± 3	11 ± 3	10 ± 3	11 ± 3	11 ± 3	13 ± 2
Cardiac output L/min	4.8 ± 1.5	4.3 ± 1.3	5.0 ± 1.5	6.0 ± 1.4	5.6 ± 1.7	5.5 ± 0.6
Cardiac index, L/min/m^2^	2.7 ± 0.9	2.4 ± 0.8	2.9 ± 0.8	3.0 ± 0.6	3.1 ± 1.6	2.8 ± 0.5
PVR, dynes/m^2^	728 ± 420	976 ± 404	596 ± 393	640 ± 214	540 ± 189	593 ± 87
MvO_2_, %	63.4 ± 9.2	59.8 ± 8.0	65.1 ± 8.9	73.3 ± 10.9	67.8 ± 7.0	56.8 ± 12.8
**Right atrial measurement**						
RA area, cm^2^	25.6 ± 9.5	27.2 ± 10.2	24.2 ± 8.4	28.3 ± 10.7	24.3 ± 9.7	28.4 ± 11.4
**Right ventricle measurements**						
RVESVi, ml/m^2^	73.6 ± 34.0	84.9 ± 31.7	63.6 ± 31.4	85.7 ± 43.9	66.7 ± 28.4	100.2 ± 27.5
RVESVi %pred	301.8 ± 141.5	69.5 ± 22.4	75.7 ± 21.6	103.2 ± 63.6	81.0 ± 28.3	76.8 ± 31.3
RVEDVi, ml/m^2^	110.8 ± 37.1	119.4 ± 34.7	99.9 ± 33.6	135.9 ± 43.5	107.4 ± 35.1	141.3 ± 38.8
RVSVi, ml/m^2^	37.2 ± 13.8	34.5 ± 10.6	36.3 ± 10.2	50.2 ± 28.8	40.8 ± 14.3	41.0 ± 16.8
RVEF, %	35.8 ± 12.8	30.1 ± 9.6	39.2 ± 12.7	38.1 ± 17.6	39.2 ± 12.2	28.8 ± 7.5
RVEF %pred	53.3 ± 18.5	45.6 ± 14.6	57.3 ± 18.3	57.8 ± 25.3	59.0 ± 18.0	44.7 ± 11.4

*MRI, magnetic resonance imaging; WHO FC, World Health Organisation functional class; PAH, pulmonary arterial hypertension; IPAH, idiopathic pulmonary arterial hypertension; HPAH, heritable pulmonary arterial hypertension; PAH-CTD, pulmonary arterial hypertension associated with connective tissue disease; PAH-CHD, pulmonary arterial hypertension associated with congenital heart disease; PoPH, portopulmonary hypertension; mRAP, mean right atrial pressure; mPAP, mean pulmonary arterial pressure; PAWP, pulmonary arterial wedge pressure; PVR, pulmonary vascular resistance; MvO_2_, mixed venous oxygen saturation; RA, right atrial; %pred, percentage predicted for age and sex; RVESVi, right ventricular end-systolic volume index; RVEDVi, right ventricular end-diastolic volume index; RVSVi, right ventricular stroke volume index; RVEF, right ventricular ejection fraction. Data are shown as mean ± SD unless otherwise stated.*

### Survival

**FIGURE 1:**
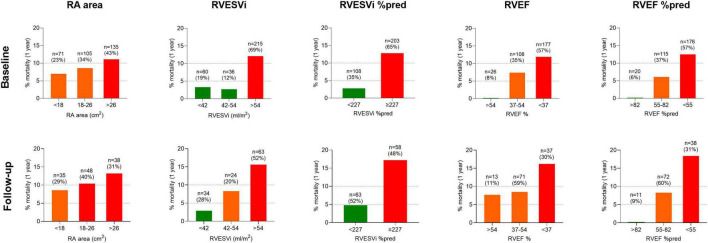
Bar charts displaying cardiac MRI RA area thresholds based on ESC/ERS guidelines and RV metrics based on published thresholds, and percentage mortality at 1-year for treatment naïve patients at (top) baseline (*n* = 311) and (bottom) follow-up (*n* = 121). MRI, magnetic resonance imaging; RA, right atrial; ESC/ERS, European Society of Cardiology and European Respiratory Society; RV, right ventricular; %pred, percentage predicted for age and sex; RVESVi, right ventricular end-systolic volume index; RVEF, right ventricular ejection fraction.

One-year following the baseline cMRI, 29 of 311 incident treatment-naïve patients had died (9.3%). Of 121 patients who underwent repeat cMRI, 13 (10.7%) died within 1-year of follow-up cMRI. Using ESC/ERS RA area risk thresholds for 1-year mortality, RA area stratified patients into intermediate and high-risk at baseline and follow-up, but was not able to identify a low-risk group either at baseline or at follow-up (Figure 1). In contrast, using previously published thresholds for RV measurements (6), right ventricular end systolic volume index (RVESVi), percentage predicted RVESVi and percentage predicted right ventricular ejection fraction (RVEF) low and high-risk groups were identified at baseline and follow-up. RVEF was able to identify low, intermediate and high-risk patients at baseline and intermediate and high-risk patients at follow-up.

**FIGURE 2:**
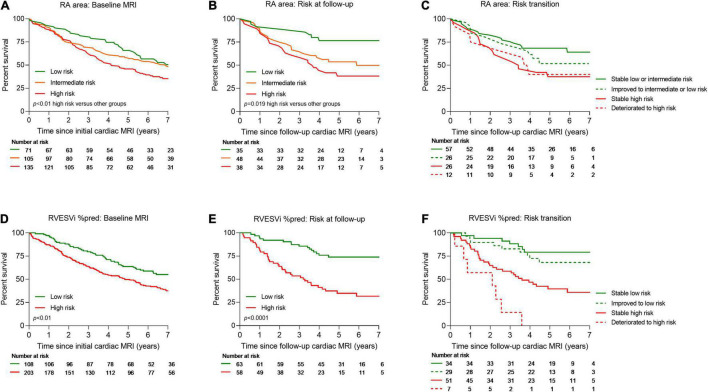
Kaplan–Meier survival curves for treatment naïve patients for RA area at baseline **(A)**, RA area at follow-up cardiac MRI **(B)**, transition of risk between baseline and follow-up cardiac MRI for RA area **(C)**, RVESVi %pred at baseline **(D)**, RVESVi %pred at follow-up cardiac MRI **(E)**, and transition of risk between baseline and follow-up cardiac MRI for RVESVi %pred **(F)**. MRI, magnetic resonance imaging; RA, right atrial; %pred, percentage predicted for age and sex; RVESVi, right ventricular end-systolic volume index.

Kaplan–Meier analysis showing survival at baseline and follow-up and risk transition at follow-up are shown in Figure 2. Baseline patients with RA area <18 cm^2^ (23% of patients) and 18–26 cm^2^ (34% of patients) had 1-year survival of 92.5% and 90.6%, respectively, while patients with RA area of >26 cm^2^ (43% patients) had 1-year survival of 87.7%. Moreover, baseline patients with percentage predicted RVESVi <227 (35% of patients) had 1-year survival of 97.5%, while patients with percentage predicted RVESVi ≥227 (65% of patients) had 1-year survival of 87.2%. Using RA area at follow-up for low risk (29% of patients) and intermediate risk (40% of patients), 1-year survival was 91.3 and 90.1%, respectively, while for high risk (31% patients), 1-year survival was 85.5%. Additionally, using percentage predicted RVESVi at follow-up low risk (52% of patients), 1-year survival was 96.9%, while for high risk (48% patients), 1-year survival was 83.5%.

A multi-variable binary logistic regression was carried out to assess the effect of age, sex, World Health Organisation functional class, percentage predicted RVESVi, RVEF, and RA area on the likelihood of 1-year mortality. It was found that age (Wald = 114.11, *p* < 0.01, odds ratio = 1.07 [95% confidence interval (CI): 1.03, 1.10]) as well as RVEF (Wald = 8.05, *p* < 0.01, odds ratio = 0.95 [95% CI: 0.92, 0.98]), but not RA area, were independent predictors of 1-year mortality.

## Discussion

Using cMRI, we have shown that measures of RV function and RA area have prognostic value, however, only measures of RV function but not current ESC/ERS RA area thresholds identify patients at low-risk of 1-year mortality.

**FIGURE 3:**
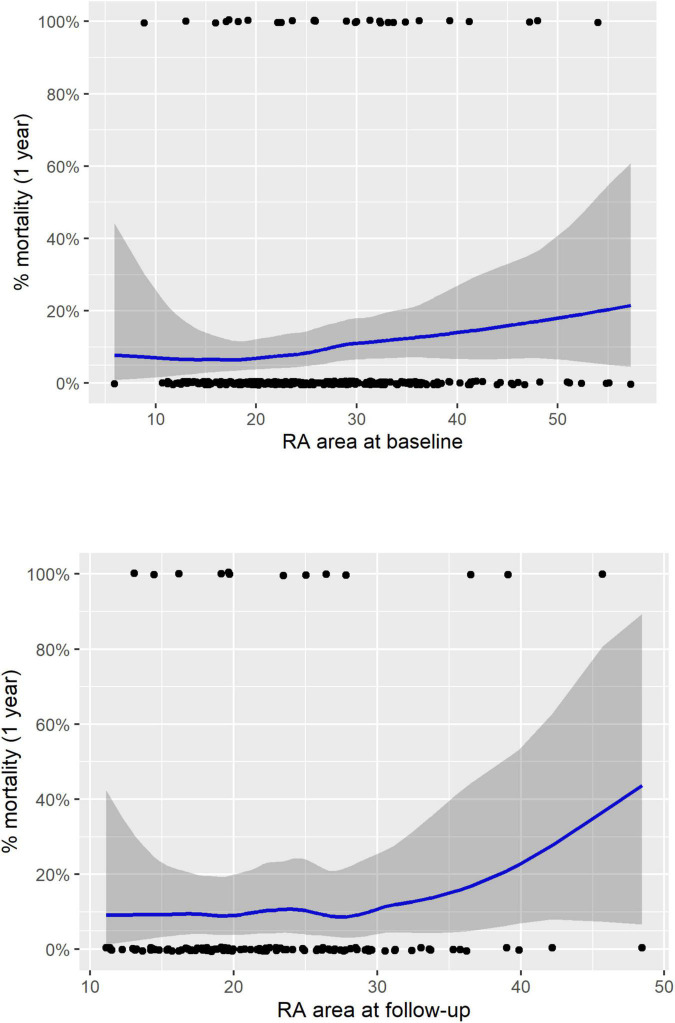
LOESS regression analysis for treatment naïve patients for RA area at baseline (top) and follow-up (bottom). RA, right atrial; LOESS, locally weighted scatterplot smoothing.

The ESC/ERS guidelines recommend that treatment is aimed at achieving and maintaining a low-risk of 1-year mortality (1) with patients remaining in an intermediate or high-risk group having a significantly higher mortality. Our study using cMRI has shown that although RA measurements can be used to identify intermediate and high-risk patients the current RA area thresholds do not allow for identification of a low-risk group. Moreover, LOESS analysis shows that the confidence intervals for mortality are large in patients who have a RA area within the normal range (Figure 3). Recent publications have highlighted other imaging parameters including measures of RV function that can identify patients at low-risk of 1-year mortality (3, 4, 6). In this study we have shown that RV measurements acquired with inclusion of trabeculations in the blood pool, can also be used to risk stratify patients. Including trabeculations in the blood pool is the most commonly used approach and is less timing consuming as it does not necessitate tracing around trabeculations. A further publication using echocardiography has shown that combining echocardiographic measures including those that assess RV function (TAPSE, tricuspid regurgitant jet grade and IVC area) stratified the risk of all-cause mortality in PAH. Inclusion of parameters such as RA area and pericardial effusion did not provide additional prognostic value (10).

This study is limited by its single centre design, nonetheless it is one of the largest cohorts of patients with PAH to have undergone cMRI. As per ESC/ERS pulmonary hypertension guidelines we measured RA area. Whether other measures of RA volume and/or function provide additional prognostic value requires further study. In addition, this study does not include a comparison with left ventricular parameters as they are less established in patients with PAH as a prognostic marker, although recent studies have demonstrated that left ventricular parameters have prognostic value (3, 11) and may have a potential role in the risk stratification of patients with PAH (6).

## Conclusion

In conclusion, this study confirms the prognostic value of RA and RV metrics using the reference standard for measures of cardiac structure and function in PAH. However, with respect to risk stratification of PAH this study highlights the need for guidelines to include measures of RV function rather than relying on RA area alone.

## Data Availability Statement

The raw data supporting the conclusions of this article will be made available by the authors, without undue reservation.

## Ethics Statement

The studies involving human participants were reviewed and approved by the Assessing the Spectrum of Pulmonary hypertension Identified at a Referral centre registry (ASPIRE, c06/Q2308/8). Written informed consent for participation was not required for this study in accordance with the national legislation and the institutional requirements.

## Author Contributions

FA, AJS, and DGK conceived the idea of the study. FA, ET, and AM analysed the MRI cardiac images. FA, RAL, and KD contributed to the statistical analysis. FA, DGK, AJS, AH, and RC prepared the figures and tables. FA, SA, CSJ, and AJS assisted with the demographic and MRI data. AL, LW, AJS, DGK, and JMW supported this study management. FA, AJS, DGK, AH, RAL, SA, KD, YS, JM, DA, SR, PG, RC, CAE, AC, AART, and AMKR interpreted the data. FA, DGK, AJS, AH, RC, AMKR, AART, AL, AM, LW, PG, AC, and CAE assisted to write the manuscript. All authors read and approved the manuscript.

## Conflict of Interest

The authors declare that the research was conducted in the absence of any commercial or financial relationships that could be construed as a potential conflict of interest.

## Publisher’s Note

All claims expressed in this article are solely those of the authors and do not necessarily represent those of their affiliated organizations, or those of the publisher, the editors and the reviewers. Any product that may be evaluated in this article, or claim that may be made by its manufacturer, is not guaranteed or endorsed by the publisher.
